# Detection of *Fusobacterium nucleatum* in Patients with Colitis-Associated Colorectal Cancer

**DOI:** 10.1007/s00284-023-03398-7

**Published:** 2023-07-19

**Authors:** Theresa Dregelies, Franziska Haumaier, William Sterlacci, Steffen Backert, Michael Vieth

**Affiliations:** 1 Institut für Mikrobiologie, Friedrich-Alexander-Universität, Staudtstr. 5, 91058 Erlangen, Germany; 2 Institut für Pathologie, Friedrich-Alexander-Universität Erlangen-Nürnberg, Klinikum Bayreuth, Preuschwitzer Str. 101, 95445 Bayreuth, Germany

## Abstract

*Fusobacterium nucleatum* is supposed to play a critical role in the development of colorectal cancer. The species has also been associated with ulcerative colitis (UC) that can progress into colorectal cancer, however, the involvement of bacteria in this process remains unclear. We analysed 177 colon biopsies obtained from patients during screening, including 20 healthy controls, 56 UC cases and 69 cases at different stages of progression to colitis-associated cancer (CAC); 32 samples of sporadic colorectal carcinoma (sCRC) were also included. The presence of *F.* *nucleatum* was detected by quantitative real-time PCR (qPCR). Our data show an association between the presence of the bacteria and the progression of carcinogenesis in UC patients. In 39.5% of CAC samples *F. nucleatum* was detected, compared to only 1.8% in UC cases. The bacteria were detected in 6.3% of samples with initial neoplastic transformation, so-called low-grade dysplasia (LGD), whereas high-grade dysplasia (HGD) resulted in 33.3% of samples positive for *F. nucleatum*. The fraction of *F. nucleatum*-positive samples from sCRC cases was 56.3%, which was not significantly different to the CAC group. We conclude that *F. nucleatum* is associated with the occurrence and progression of colon carcinogenesis, rather than with UC itself.

**Supplementary Information:**

The online version of this article contains supplementary material available 10.1007/s00284-023-03398-7.

## Introduction

Ulcerative colitis (UC) is a common form of inflammatory bowel disease (IBD) that is characterized by chronic inflammation of the colon mucosa. The inflammatory process starts from the rectum and spreads continuously to the colon and can even reach the terminal ileum. Clinical characteristics of UC include abdominal pain, often accompanied by bloody diarrhoea [1]. In 2017, the worldwide prevalence of UC was reported as 84.3 per 100,000 people, with by a death rate of 0.51 per 100,000 [2]. The most recent data from Germany are from 2019, when the UC incidence rate was 36 per 100,000, with a prevalence of 529 per 100,000 [3].

Patients suffering from UC have an increased risk of developing colorectal cancer (CRC) [4]. The carcinogenesis of this colitis-associated cancer (CAC) follows a typical sequence of inflammation-dysplasia-carcinoma [5]. The progression starts with the onset of UC-induced inflammation of the colon mucosa, which over time may lead to neoplasia. Precancerous stages of low-grade dysplasia (LGD) and high-grade dysplasia (HGD) can be recognized [6], although occasionally UC can directly progress towards CAC without a precancerous stage [7].

The distinction between CAC that is directly caused by UC and CRC that is independent of UC but initiated by sporadic somatic mutations (sCRC) can be difficult, based on histology only; likewise, the corresponding precancerous lesions are not always easy to diagnose [8]. However, a correct diagnosis is important as it has a significant impact on treatment. If an area in the colon is diagnosed with sporadic lesions, its endoscopic removal is considered curative and in CRC patients without UC a partial colectomy is considered, while CAC or HGD is recommended to be treated by proctocolectomy [9]. So far, the exact molecular mechanisms of pathogenesis for the development of CAC have not been clarified, however, it has been suggested that a genetic predisposition of the host, diet and lifestyle, and the gut microbiome may all play a role.

The human intestine is densely populated with approximately 10^12^ microorganisms per gram content [10]. Some components of this microbiota can be involved in inflammatory processes or can cause DNA damage leading to cell death, and as such they may have an impact on CRC development [11]. In 2012, two independent research groups described an increased occurrence of *Fusobacterium nucleatum* in cancerous intestinal tissue [12, 13], and since then, the impact of *F. nucleatum* in the development or progress of CRC has been substantiated [12–17]. However, the underlying mechanisms that lead to the progression of carcinogenesis caused by this bacterial species remain unclear. It has been suggested that *F. nucleatum* might cause damage to the epithelial barrier of the colon [18], induce DNA damage of intestinal mucosal cells [19] or lead to dysbiosis of the gut microbiota [20].

*F. nucleatum* is a Gram-negative, obligate anaerobe bacterium typically residing in the oral cavity [21]. It is considered pathogenic when it is involved in dental plaque formation, where it is able to attract other bacterial species [22]. The bacteria have occasionally been detected in various other organs as well, including in the placenta and in foetal tissue [23], in the brain [24] and in the liver [25]. In mice, oral fusobacteria were hematogenously transferred to the placenta and this was associated with stillbirth [26]. When *F. nucleatum* is present in the colon, this is associated with IBD and this corelated with its invasive potential [27].

The pathogenicity of *F. nucleatum* is related to its ability to attach to and invade into epithelial cells [27, 28], enabled by virulence factor FadA that is expressed on the bacterial surface [29, 30]. FadA acts as an adhesin and binds to the protein E-cadherin present on host cells at adherens junctions. This activates transcription factor β-catenin signalling, a pathway that is also involved in cell proliferation during carcinogenesis. It has been shown that FadA levels are significantly increased in colon tissue from patients with adenomas and adenocarcinomas, and higher FadA levels in CRC tissue correlate with increased expression of oncogenic and inflammatory genes [14]. Increased expression of cytokines such as IL-6 and IL-1β in patients with an *F. nucleatum* infection was described that could drive the local inflammatory response [18, 31, 32].

Although a number of studies reported a correlation between the occurrence of *F. nucleatum* and CRC [33–36], published data on its involvement in IBD or UC have been contradictory. A Canadian study described the occurrence of *F. nucleatum* in 50% of IBD patients and suggested that *F. nucleatum* could serve as a possible biomarker for IBD [27]. The bacteria were also detected in 39% of UC patients from China with a similar detection rate of 37.14% in CRC patients, indicating an association between *F. nucleatum* and UC as well as CRC [28]. In contrast, a Japanese study found *F. nucleatum* in only 6.3% of the analysed UC patients [37]. It was reported that the number of colonic *F. nucleatum* bacteria correlates with a shorter survival in CRC cases [17] and that high numbers aggravate the course of UC by damaging the intestinal barrier and promoting inflammation [18]. Furthermore, *F. nucleatum* was shown to be associated with resistance to chemotherapy [38, 39].

This conflicting literature concerning the association of *F. nucleatum* with UC and CRC led to the present study, which aimed to establish the occurrence of qPCR-detected *F. nucleatum* in German patients with UC, with emphasis on the different stages of CAC carcinogenesis.

## Material and Methods

### Patient Groups and Sample Collection

In this study, consecutive, retrospective samples of 177 patients that were treated endoscopically or surgically at the Klinikum Bayreuth between 2006 and 2020 were analysed. Biopsies and the surgical resection material were sent to the Institute of Pathology of the Klinikum Bayreuth in Germany for histopathological assessment. UC-associated dysplasia (LGD, HGD) and cancerous lesions (CAC, sCRC) were diagnosed according to common criteria and guidelines [9, 40]. All diagnostic results were confirmed by two independent pathologists with consistent outcomes. The study was approved by the ethics committee of the University Bayreuth (#O 1305/1-GB).

The biopsies (*N* = 177) were fixed in 4% neutral pH-buffered formalin. Tissue samples were dehydrated and paraffinized using a HistoCore PELORIS 3 (Leica Biosystems, Germany). Formalin-fixed paraffin-embedded (FFPE) blocks were cut into 4-µm-thick slices, followed by hematoxylin–eosin staining carried out on a Tissue-Tek Prisma (Sakura Finetek, Japan) for histopathological diagnosis. According to the diagnosis, the patients were categorized in six groups, with group 1: healthy colon (*n* = 20), group 2: UC (*n* = 56), group 3: LGD (*n* = 16), group 4: HGD (*n* = 15), group 5: CAC (*n* = 38) and group 6: sCRC (*n* = 32).

The FFPE blocks were microdissected to 5 µm and genomic DNA was extracted with the Maxwell LEV Blood DNA Kit (Promega) on a Maxwell 16 instrument. The DNA was quantified with the QuantiFluor dsDNA System Kit (Ref. E4871) in combination with a Quantus Fluorometer (Promega).

As a positive control, a bacterial strain of *Fusobacterium nucleatum, subsp. nucleatum* (DSM 15643) was obtained from the Leibnitz-Institute, (Deutsche Sammlung von Mikroorganismen und Zellkulturen GmbH, Braunschweig, Germany). The freeze-dried strain was resolved in thioglycollate medium enriched with vitamin K1 and Hemin (Becton Dickinson Ref. 221,788) by colleagues from the Institute for Laboratory Medicine (ILM, Klinikum Bayreuth) and grown under anaerobe conditions using GasPak EZGas (Becton Dickinson, Ref. 260,683) for 48 h at 35 °C. The culture was then incubated on Schaedler Agar with vitamin K1 and 5% sheep blood (Becton Dickinson, Ref. PA-254084). Colonies were transferred to 1 mL 4% formalin and centrifuged for 10 min at 300 RPM. The pelleted bacteria were embedded in paraffin and treated as the FFPE blocks as described above to extract bacterial DNA. All DNA preparations were stored at − 20 °C.

### Quantitative Real-Time Polymerase-Chain Reaction (qPCR)

A unique sequence within the *F. nucleatum nusG* gene was used as the target sequence for qPCR [12]. The used primer sequences were taken from the literature [34], with forward primer 5'-CAACCATTACTTTAACTCTACCATGTTCA and reverse primer 5'-GTTGACTTTACAGAAGGAGATTATGTAAAAATC. The human gene *slco2a1* was used as the internal control, with forward primer 5'-ATCCCCAAAGCACCTGGTTT and reverse primer 5'-AGAGGCCAAGATAGTCCTGGTAA. Reactions were performed in 20 µL containing 1 × LightCycler FastStart DNA Master SYBR Green I Kit (Roche, Penzberg), 3 mM MgCl_2_ and 0.1 µM of each primer in nuclease free, PCR-grade water (Roche, Penzberg). As a template, 0.3–100 ng of FFPE-isolated DNA was used. The positive control contained 20 pg of isolated *F. nucleatum* DNA and nuclease free, PCR-grade water was used as the negative control. The qPCR reactions were performed with a Cobas LightCycler z480 (Roche, Penzberg) using the following conditions: denaturation for 10 min at 95 °C, 50 cycles of 95 °C for 5 s, 65 °C for 10 s and 72 °C for 6 s, followed by a temperature gradient from 37 to 95 °C with a ramp rate of 0.06 °C/s for melting curve analysis. Melting curves were evaluated with the LightCycler 480 SW Software version 1.5.1.62.

Produced amplicons were checked by agarose gel electrophoresis to confirm their length of 112 bp and 74 bp for *nusG* and *slco2a1*, respectively. The correctly sized bands were excised, and the amplified DNA was purified with the QIAquick Nucleotide Removal Kit (Qiagen, Hilden) and sequenced (Eurofins GATC, Köln) using the forward primers of *nusG* or *slco2a1*, respectively. The obtained sequences were compared to the corresponding GenBank entries (accession number of *nusG* from *F. nucleatum*: AE009951.2; accession number of human control gene *slco2a1*: NC_000003.12) using BLAST.

### Establishment, Precision and Limit of Detection by qPCR

To verify that the *nusG* and *slco2a1* targets are suitable for simultaneous amplification in a multiplex qPCR, the melting temperatures of both products were determined in separate reactions first, for which 20.4 ng isolated *F. nucleatum* DNA and 24 ng of isolated human DNA, respectively, was used. Reactions were carried out in quadruples to assess intraassay replication. For detection of interassay precision, both products were co-amplified in three independent experiments using the same DNA template samples.

The limit of detection (LOD) of the qPCR analysis was determined with serial dilutions of isolated *F. nucleatum* DNA (range 6.7–0.2 ng) that were complemented with a constant amount (24 ng) of human DNA to create a consistent background for all reactions (Table S1).

### Statistics

Fisher’s exact test was used to investigate correlations between the detection of *F. nucleatum* and the different stages of carcinogenesis. For groups with a sample size > 5, the Pearson’s Correlation Coefficient was used. Statistical analyses were carried out SPSS Statistics Version 23.0.0.0. Power analysis was conducted with G*Power Version 3.1.9.7 (One-sided Fisher’s exact test, power = 0.80, *α*-error: 0.05) to detect the required sample sizes of all six groups (Table S2).

## Results

### qPCR Design and Optimization

The presence of *F. nucleatum* in biopsies from patients with UC at different stages of carcinogenesis was investigated by qPCR. For this, we performed multiplex qPCR to simultaneously amplify the *nusG* gene as a qualitative detection marker for the presence of *F. nucleatum* and of *slco2a1*, a human gene that served as the internal control. First, we established the melting curves of both products in separate reactions. Based on four replicate experiments, the amplicon of *nusG* had a melting temperature (Tm) of 77.67  ± 0.10 °C, whereas the Tm of the *slco2a1* product was 80.29 ± 0.10  °C, indicating a high intraassay precision. The results of the four replicates are summarized in Table S3. The difference of 2.62 °C between the Tm of both products was considered sufficient for a multiplex PCR approach. To determine the interassay precision, *nusG* and *slco2a1* were co-amplified in triplicate, using the same DNA as the template. In this multiplex assay, we observed slightly lower melting temperatures for both products compared to separate amplification: the Tm of the *nusG* product was now 76.66 ± 0.04 °C and the Tm of the *slco2a1* amplicon was 80.17 ± 0.10 °C (see Table S4 for the individual experiments). Both melting temperatures showed high reproducibility, and their difference had even increased, to 3.51 °C on average, which was sufficient for multiplex amplification and detection.

Because our aim was to detect *F. nucleatum* in samples containing human DNA, a high background of the latter could be expected. Therefore, we determined the limit of detection (LOD) of *F. nucleatum* DNA by spiking a dilution series of bacterial DNA in the presence of a constant, high background of human DNA (Table S1). Even at the lowest tested concentration, corresponding to a bacterial to human DNA ratio of 0.8%, *F. nucleatum* DNA was still detected. This corresponded with a limit of detection of 10 pg µl^−1^ for *F. nucleatum*.

**Fig. 1:**
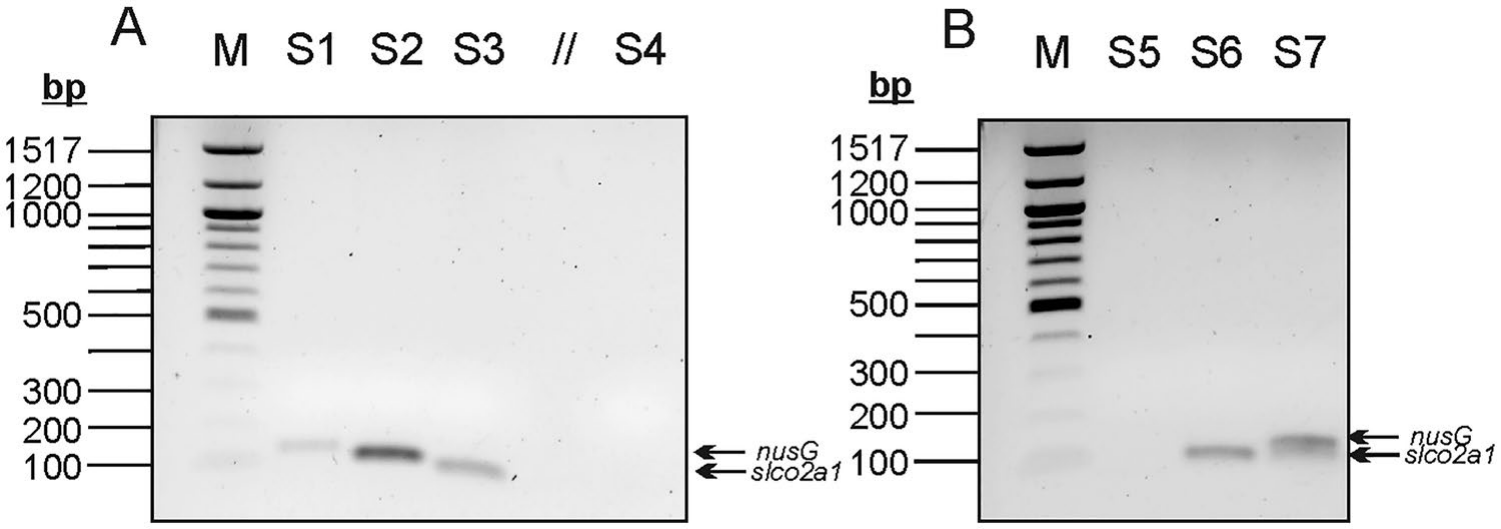
Agarose gel electrophoresis of the qPCR products obtained during separate amplification (**A**) and co-amplification (**B**) of bacterial *nusG* and human *slco2a1*. **A** M: 100 bp size marker, S1 and S2: *F. nucleatum* DNA amplified with *nusG* primers (positive control), S3: human DNA amplified with *slco2a1* primers, S4: negative control in the presence of both primer pairs. Lane 5 is empty. **B** M: 100 bp size marker, S5: amplification of an *F. nucleatum*-negative human sample with *nusG* primers only, S6: co-amplification of an *F. nucleatum*-negative sample with both primer pairs, S7: co-amplification of an *F. nucleatum*-positive sample with both primer pairs. Arrows indicate the positions of the *nusG* and *slco2a1* amplicons

The PCR products obtained in the single and multiplex assays were validated by gel electrophoresis (Fig. 1). This resulted in amplicons sized 74 bp and 112 bp, for *slco2a1* and *nusG* amplicons, respectively.

To confirm that the amplicons were indeed produced from the target genes, the bands of samples S1 and S3 were excised from the agarose gel (Fig. 1) and after purification the DNA was sequenced using the corresponding forward primers utilized for qPCR amplification. When the DNA sequence obtained from sample S1 was compared to a GenBank entry of *nusG* (Accession number AE009951.2) it aligned with 92% identity. Alignment of the sequence from sample S3 with a GenBank entry of *slco2a1* (Accession number NC_000003.12) revealed a sequence identity of 95%. These results proved specificity of the used primers for both genes, producing amplicons of the correct length for both targets.

**Fig. 2:**
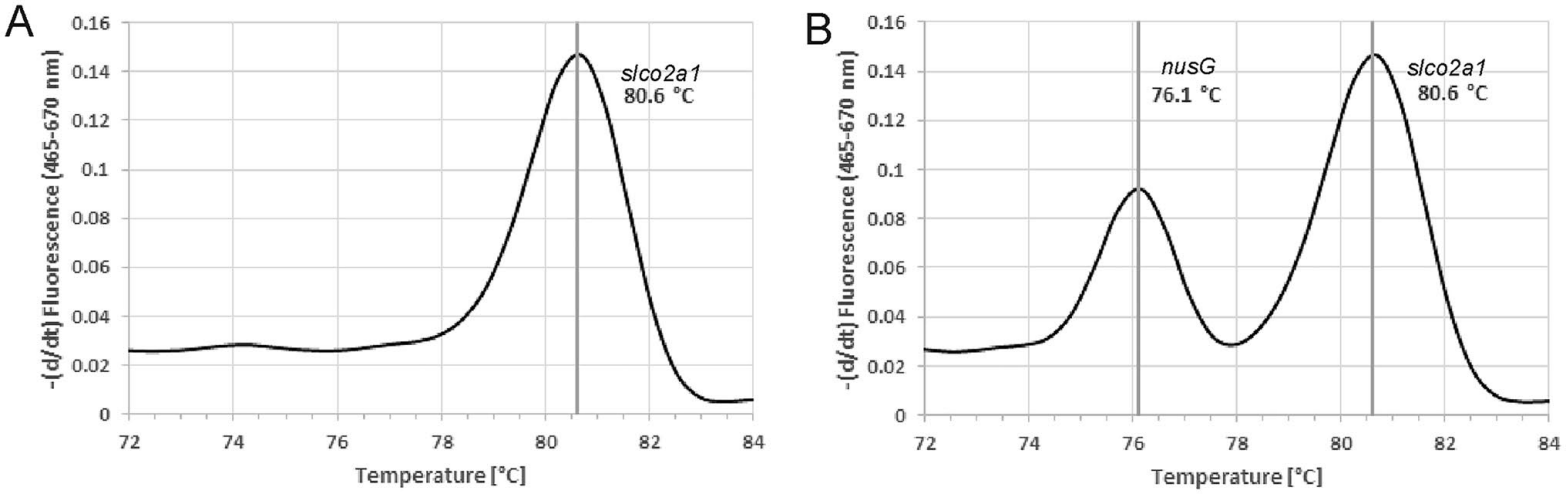
Melting point analysis in a multiplex qPCR for detection of *F. nucleatum* in the presence of human DNA in FFPE samples. The presence of bacteria was indicated by amplification of the *F. nucleatum*-specific *nusG* gene with a melting temperature of 76.21 °C ± 0.53 and the absence of PCR inhibition was confirmed with the internal standard of the human *slco2a1* gene (melting temperature: 80.32 °C ± 0.33). **A** shows the melting curve obtained with an *F. nucleatum*-negative sample and **B** that of a *F. nucleatum*-positive sample

Melting point analysis was next performed for all 177 human samples by multiplex PCR. Examples of the melting curves obtained with *F. nucleatum*-positive and *F. nucleatum*-negative samples, respectively, are shown in Fig. 2. Based on the mean results obtained from all samples, a sample was considered positive for the presence of *F. nucleatum* if a peak was present at 76.21 °C ± 0.53 in addition to the peak from the internal control of human *slco2a1* present at 80.32 °C ± 0.33. Isolated *F. nucleatum* DNA was included as positive control in each amplification series. This resulted in mean Tm values of 77.71 °C ± 0.17. These data imply consistent and reproducible melting temperatures throughout all samples. A total of 40 samples were found positive for the presence of *F. nucleatum*, while 137 samples were negative.

### Prevalence of *F. nucleatum* in Colon Biopsies from Patient Groups

**Fig. 3:**
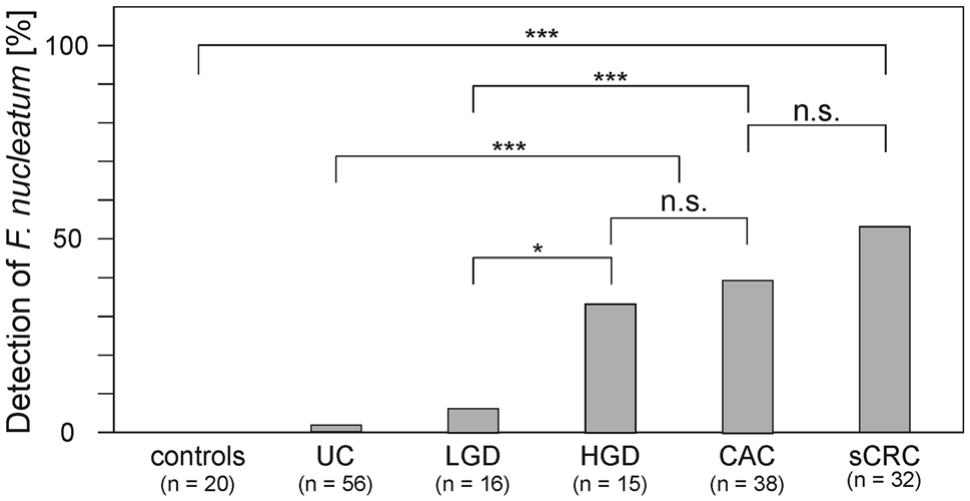
Prevalence of *F. nucleatum* in human colon biopsies. The percentage of positive samples is plotted for each group, with the number of samples per group indicated. ***p ≤ 0.001, ** *p* ≤ 0.01, * *p* ≤ 0.05, n.s. not significant

The results of the qPCR were analysed per patient group, to investigate the association between the presence of *F. nucleatum* and either UC, CAC or sCRC (Table 1). The melting curve data are summarized per group in Table S5. The bacteria were not detected in samples from healthy controls and their prevalence strongly differed between groups of patients with different pathology. Whereas *F. nucleatum* was only detected in 1.8% of patients diagnosed with UC (*n* = 56), one-third or more of patients with HGD (*n* = 15) or CAC (*n* = 38) were found positive (Table 1). Figure 3 summarizes the prevalence of *F. nucleatum* for each group. Compared to group 2 of UC patients, the higher prevalence of *F. nucleatum*-positive samples in group 4 (HGD) and 5 (CAC) was highly significant (*p* = 0.001 and 0.000, respectively). Significant differences were also detected between group 3 (LGD) and either group 4 or 5 (*p* = 0.021 and  0.000, respectively). The results implicate that patients presenting with HGD already more often had *F. nucleatum* in their colon than patients with UC or LGD. The prevalence of *F. nucleatum* in patients with HGD and CAC was comparable (*p* = 0.761). Interestingly, the bacteria were detected at a similar prevalence in CAC and sCRC samples (*p* = 0.230).

**Table 1:** The presence or absence of *F. nucleatum* detected by qPCR in patient six groups with different pathologies, and subgroup analysis for sex and age

Group	Sex	Sample size	Age (years)	Detection of *F. nucleatum*		
				Positive	Negative	% positive
All samples	F + M	177	all	40	127	34
Group 1 Healthy controls	F + M	20	21 to > 80	0	20	0
Group 2 UC	F + M	56	< 21 to 80	1	55	1.8
	Female	26	21 to 40		8	
			41 to 60		9	
			61–80	1	8	
	Male	30	< 21		3	
			21–40		10	
			41–60		11	
			61–80		6	
Group 3 LGD	F + M	16	41 to > 80	1	15	6.3
	Female	4	61–80		3	
			> 80		1	
	Male	12	41–60		5	
			61–80	1	6	
Group 4 HGD	F + M	15	21 to > 80	5	10	33.3
	Female	6	41–60		2	
			61–80	2	1	
			> 80	1		
	Male	9	21–40		1	
			41–60	1	1	
			61–80	1	5	
Group 5 CAC	F + M	38	21 to > 80	15	23	39.5
	Female	13	21–40	1		
			41–60	2	5	
			61–80	2	2	
			> 80		1	
	Male	25	21–40		2	
			41–60	6	6	
			61–80	4	7	
Group 6 sCRC	F + M	32	21 to > 80	18	14	56.3
	Female	20	41–60	3	1	
			61–80	7	6	
			> 80	3		
	Male	12	21–40	1		
			41–60	1	3	
			61–80	2	4	
			> 80	1		
All groups	Female	79		22	57	28
All groups	Male	98		18	80	18
All groups		3	< 21	0	3	0
All groups		31	21–40	2	29	6
All groups		62	41–60	13	49	21
All groups		73	61–80	20	53	27
All groups		8	> 80	5	3	63

**Fig. 4:**
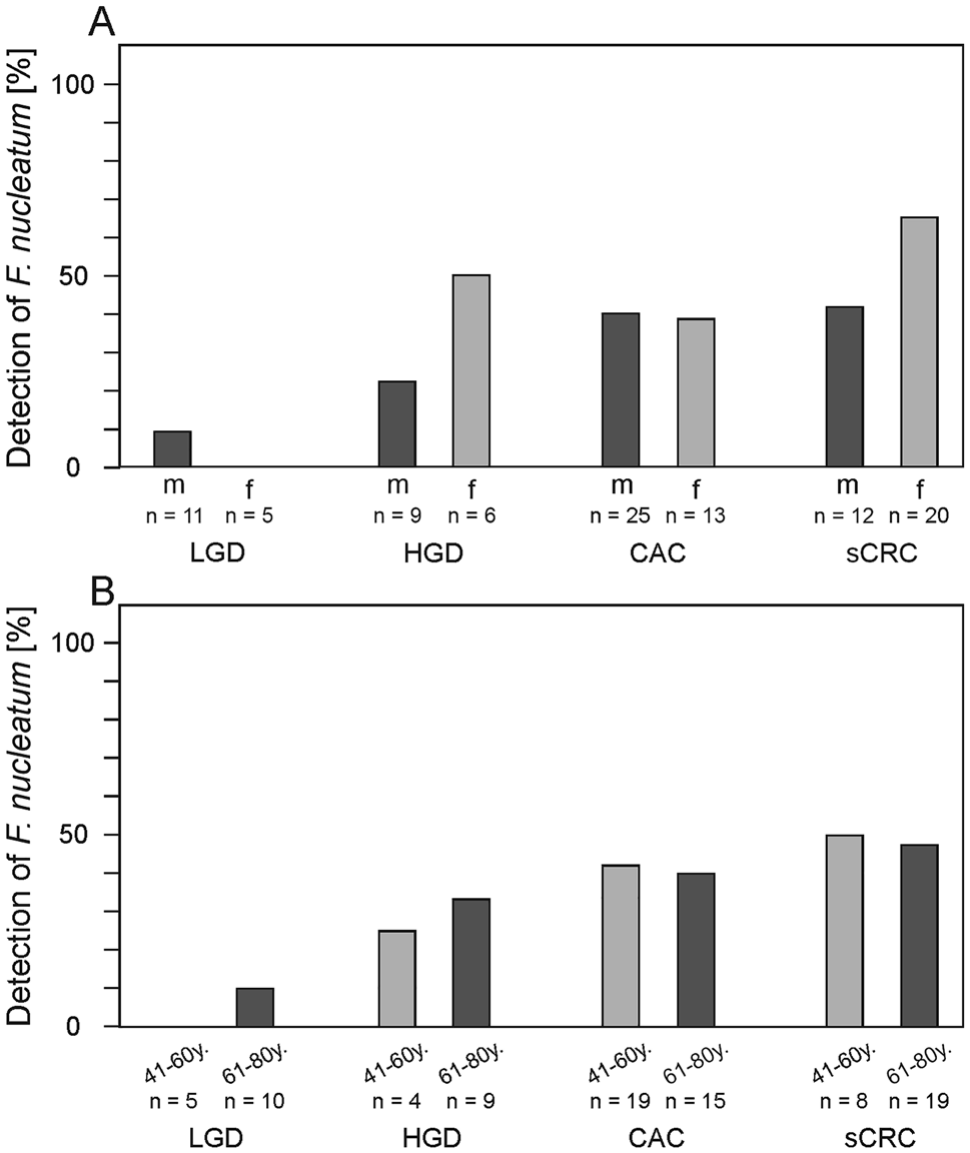
Prevalence of *F. nucleatum* in subgroup analysis for sex (**A**) or age (**B**). Due to a small sample size, Groups 1 (healthy controls) and 2 (UC) are not shown, and age groups < 40 years and > 80 years are not shown in panel B

The results for each group were analysed for patient’s sex and age (Table 1). Whereas 28% of female patients were found positive, only 18% of male patients were positive (Table 1). Thus, females were about 1.5 times more likely to be positive for the bacteria than males, but this difference was not significant (*p* = 0.206). The results for sex within the patient groups are summarized in Fig. 4. Since no positive samples were obtained from the healthy controls of group 1 and only one of the 56 patients in group 2 was positive for *F. nucleatum*, these two groups were omitted here. Concentrating on groups 3 to 6, a total of 45% female and 55% male patients were found positive for *F. nucleatum*. In 28% of samples obtained from females and in 19% of samples from males, *F. nucleatum* was detected. The most remarkable differences between male and female patients were seen in group 4 (HGD with 50% prevalence in females versus 22% in males) and group 6 (sCRC, 65% versus 42%, Fig. 4A), but this difference was not statistically significant (HGD: *p* = 0.329; sCRC: *p* = 0.277). Interestingly, about half of the analysed patients for both sexes in group 5 presenting with CAC (male: 40%, 10/25; female: 39%, 5/13) were infected with *F. nucleatum* (*p* = 1.000), which differs from the results obtained for groups 4 and 6. A comparison of patients with regard to the sex in groups 5 and 6 revealed no statistical relevance (female: *p* = 0.169, male: *p* = 1.000, respectively). Together, these results underline that the infection is not correlating with the sex nor with the type of cancer in the investigated patients.

Since patients with UC are usually quite young, with an initial manifestation of UC between the age of 20 and 30 [41], we further analysed the data according to the patients’ age. For this, the patients were divided into five different subgroups of < 21 years (y), 21–40 years, 41–60 years, 61–80 years and > 80 years. The highest prevalence of *F. nucleatum* was found in samples from patients 41–60 years (21%) and 61–80 years (27%), and as many as 5 of 8 samples obtained from > 80-year-old patients were positive (Table 1). Ignoring the clinical presentation, a statistically significant difference was found in *F. nucleatum* prevalence between patients up to 40 y and those older (*p* = 0.011).

Figure 4B summarizes the data for subgroups 41–60 years and 61–80 years only per clinical manifestation; the other age subgroups had too few samples. A slight increase in prevalence was observed, in both age groups, going from LGD to HGD, CAC and then sCRC. While none of the patients with LGD were positive for *F. nucleatum*, that fraction increased to 25% (1/4) and 42% (8/19) in HGD and CAC samples, respectively (*p* = 0.444, 0.130). A similar increase was found in the age group of 61–80 years, where the fraction of positive patients was the lowest in group 3 with LGD (10%, 1/10) and the highest (40%, 6/15) in group 5 with CAC, which was not statistically different (*p* = 0.303, 0.107, respectively).

These findings all suggest that *F. nucleatum* prevalence increases the closer the patient is to the diagnosis of CAC. We point out that, of all clinical groups, samples from sCRC cases resulted in the highest prevalence of *F. nucleatum* (Fig. 3), for both sexes (Fig. 4A) and in three age subgroups: sCRC samples resulted in a prevalence of 50% (4/8) at 21–40 years, 47% (9/19) at 41–60 years and all four samples from > 80 years patients were positive. The frequencies of positive samples were similar between the CAC and sCRC groups, not resulting in a significant difference (Fig. 3), and the distribution of the positive samples in the age groups 41–60 years and 61–80 years within the groups 3–5 was similar.

## Discussion

*F. nucleatum* can be involved in opportunistic infections and a role in CRC has been speculated, however, the species is a common member of the oral microbiota and can also have benign relationships with the host [21]. A recent study reported the association of *F. nucleatum* DNA in stool samples from IBD patients [19]. In mouse models, *F. nucleatum* had an impact on the intestinal inflammation for dextran sodium sulphate (DSS)-induced colitis, achieved by causing damage of the epithelial barrier [18, 42]. In our study, colon biopsies of 177 German patients presenting with various clinical pathologies were examined for the presence of *F. nucleatum*. Our data show that the incidence of *F. nucleatum* is not only increased in CAC cases compared to controls, but also in the precancerous stages LGD and in particular HGD. The prevalence of the bacteria was significantly higher in HGD and CAC compared to tissue samples from cases of LGD or UC. From this, we conclude that *F. nucleatum* infection does not represent an early event in CAC carcinogenesis. This implies that *F. nucleatum* may not be useful as an early diagnostic marker for CRC development, however, its presence may indicate a progression in malignant transformation. A metagenomics study analysing human faeces indicated that *F. nucleatum* possibly acts as a key factor for the initiation of disorders, implying that this bacterium might be a useful early biomarker for CRC development [43].

Our results from the studied German cohort of patients corroborated data from international studies. It has been demonstrated in patients from North America [12, 34], South America [44], Asia [35, 36, 45] and Europe [33, 46] that *F. nucleatum* is overabundant in tumorous colon samples from tissue or stool, compared to adequately matched healthy controls. Still, there are differences within literature reports in the frequency of *F. nucleatum* detection. Whereas a Chinese study identified 87.13% of CRC samples positive for the bacteria [36], a Japanese study [47] and two studies from the USA reported positivity for CRCs in only 8.7% and 13%, respectively [17, 34]. Compared to those findings, our data included 70 cases of CRC (CAC and sCRC combined) of which 47.1% (33/70) were found positive, which places our German cohort somewhere in the middle of the reported values from other studies. Differences between studies may be due to the method of detection and the nature of the samples. A recent study demonstrated the impact of the sample type on *F. nucleatum* detection by qPCR [48]: while *F. nucleatum* was detected in 23% of CRCs from fresh-frozen tissues, only 5.8% of CRC-derived FFPE tissues were positive. In this respect, our reported prevalence, detected in FFPE samples, is eight times higher, possibly related with the high sensitivity and low limit of detection that was achieved.

A Chinese study reported a prevalence of *F. nucleatum* in only 6.3% (4/64) of examined UC samples [36]. Our data confirm that the bacteria are not common in the colonic mucosa of patients with UC, as we detected it in less than 2% (1/56) of the UC samples. The single positive sample was obtained from a patient suffering from UC at a highly active state, which could mean that *F. nucleatum* had been attracted by the active inflammation of this area, or *F. nucleatum* had induced the active inflammation.

Considering the initial stage of neoplasia (LGD), we observed a slightly higher prevalence of the bacteria (6.3%, 1/16) than in the UC group, but the difference was not significant. There was, however, a significant increase in the number of positive samples in HGD cases, which represent the precancerous stage to CAC (Fig. 3). We conclude from this that the presence of *F. nucleatum* is more likely associated with the progression of carcinogenesis, rather than the inflammation of UC itself. It is still possible that this species directly or indirectly triggers the development of carcinoma, perhaps at a later stage than LGD. This is supported by the results we obtained from sCRC samples, which produced the highest prevalence (56.3%) of *F. nucleatum*. Because this type of carcinoma does not involve chronic inflammation of the colon, the high prevalence of the bacteria here indicates that UC is not required for bacterial colonization by *F. nucleatum* in a cancerous colon. Rather, the fact that *F. nucleatum* can be detected in increasing fractions during progression to CRC suggests that *F. nucleatum* is involved in carcinogenesis. However, it remains unclear, to what part the bacterium contributes to the pathogenic cascade leading to cancerogenesis, and to reveal this, future studies are required.

The observed increase in the fraction of *F. nucleatum-*positive samples within the cascade of progression towards CAC implicates that the pathogen may represent a central factor within the pathways leading to carcinogenesis. A previous European study analysing CRC in general and colorectal adenoma (CRA) detected *F. nucleatum* with results comparable to our data [33]. While the presence of *F. nucleatum* was significantly higher in CRC and HGD samples compared to normal tissue, this was not found in CRA samples [33]. Similar results were also obtained in an independent study that involved a smaller sample size, but again reported a higher prevalence of *F. nucleatum* in CRCs compared to matching healthy individuals [49]. That study focussed on CRA as the precancerous stage of CRC, and the authors postulated that patients with high abundance of *F. nucleatum* are more likely to develop CRA, indicating an association of these bacteria with development of CRC [49].

A limitation of our study is the relatively small number of patients with LGD or HGD in our cohort, and further analyses should be conducted with expanded sample sizes for each group. This would increase the statistical power of analysis, thereby possibly fortifying our conclusion that *F. nucleatum* is probably not a suitable early diagnostic marker for cancer development.

Methods based on qPCR are commonly used for pathogen detection, as it can reach high sensitivity and specificity. The usage of intercalating dyes such as SYBR Green results in a cost-effective method, but it can lead to unspecific binding to dsDNA. In addition, unspecific amplification of by-products can contribute to the fluorescent signal. To avoid erroneous results, we applied strict measures to avoid contamination, included an internal standard to detect PCR inhibition and verified the produced amplicons to ensure the target sequences were correctly detected. When such measures are taken, qPCR is a reliable detection method. Nevertheless, the results strongly depend on the type of material used. DNA isolated from fresh-frozen tissue is more suitable for qPCR analysis than DNA from FFPE material [48]. Unfortunately, in pathology FFPE represents the standard tissue type, as it clearly is advantageous with easy sample handling and suitability for long-time storage. Although our qPCR method for FFPE samples was highly sensitive, with a detection limit of 10 pg µl^−1^ of *F. nucleatum* DNA, alternative methods should be considered, such as droplet digital PCR (ddPCR) for identification of low-abundant targets. That method was recently shown to have an even higher sensitivity than to qPCR for the detection of *F. nucleatum* and it can be applied FFPE tissue [50]. Metagenomic sequencing can also be used for the detection of the bacteria [38] and this has the advantage to determine the complete microbiome of a sample, but the costs, time requirement and downstream bioinformatic analysis still hamper routine application. For the time being, that leaves qPCR as a suitable method of detection of *F. nucleatum* in FFPE samples.

## Conclusions

*F. nucleatum*, a Gram-negative bacterium that typically resides in the oral cavity, has been implied to contribute to the development of CRC. While the underlying mechanisms have still not been resolved, it is undisputed that *F. nucleatum* is overabundant in inflammatory intestinal lesions. We investigated the presence of these bacteria by qPCR in inflammatory colon biopsies from UC cases and at different stages of carcinogenesis. Our data confirm an increased prevalence of *F. nucleatum* in lesions with sCRC, CACs and colitis-associated HGD compared to tissues from a non-inflamed colon and from cases presenting with UC only. From this, we conclude that a higher prevalence of *F. nucleatum* is associated with advancing cancerous development, but not with UC itself.

## Supplementary Information

Below is the link to the electronic supplementary material.

Electronic supplementary material 1 (DOCX 24 kb)

## Data Availability

Not applicable.
