# Supplementary material for: Detection of Fusobacterium nucleatum in Patients with Colitis-Associated Colorectal Cancer
Source: Curr Microbiol. 2023 Jul 19;80(9):293. doi: 10.1007/s00284-023-03398-7 (PMC10356651; doi:10.1007/s00284-023-03398-7)
Supplement: Supplementary file 1 — Electronic supplementary material 1 (DOCX 24 kb) [file 284_2023_3398_MOESM1_ESM.docx]

**Supplementary tables**

**Detection of *Fusobacterium nucleatum* in patients with colitis-associated colorectal cancer**

Theresa Dregelies^1,2^, Franziska Haumaier^2^, William Sterlacci^2^, Steffen Backert^1^, Michael Vieth^2,*^

^1^Institut für Mikrobiologie, Friedrich-Alexander-Universität, Staudtstr. 5, 91058 Erlangen, Germany

^2^Institut für Pathologie, Friedrich-Alexander-Universität Erlangen-Nürnberg, Klinikum Bayreuth, Preuschwitzer Str. 101, 95445 Bayreuth, Germany

^*^Corresponding author: Prof. Dr. med Michael Vieth, E-Mail: michael.vieth@fau.de, ORCID: 0000-0002-4336-7721, Tel.: +49 921/400-5602, Fax: +49 921/885602

**Table S1:** Serial dilutions of F. nucleatum DNA for the detection of its limit of detection.

| Sample name | Fn 0,337 | Fn 0,286 | Fn 0,255 | Fn 0,204 | Fn 0,179 | Fn 0,153 | Fn 0,128 | Fn 0,102 | Fn 0,010 |
| --- | --- | --- | --- | --- | --- | --- | --- | --- | --- |
| Amount of *F. nucleatum* DNA [ng] | 6.7 | 5.7 | 5.1 | 4.1 | 3.6 | 3.1 | 2.6 | 2.0 | 0.2 |
| Amount of human DNA [ng] | 24.0 | 24.0 | 24.0 | 24.0 | 24.0 | 24.0 | 24.0 | 24.0 | 24.0 |
| Ratio of *F. nucleatum* DNA to human DNA [%] | 27.9 | 23.8 | 21.3 | 17.1 | 15.0 | 12.9 | 10.8 | 8.3 | 0.8 |

_Fn =_ *_F. nucleatum_*

**Table S2**: Results of power analysis. Fields highlighted in grey represent groups of adequate sample size.

|  | Control | UC | LGD | HGD | CAC |
| --- | --- | --- | --- | --- | --- |
| UC | 373 |  |  |  |  |
| LGD | 106 | 261 |  |  |  |
| HGD | 19 | 21 | 32 |  |  |
| CAC | 13 | 17 | 23 | 779 |  |
| sCRC | 9 | 10 | 12 | 66 | 119 |

**Table S3:** Detection of melting temperatures of amplified fragments of nusG and slco2a1 in separate experiments.

|  | Tm*_nusG_* [°C] | Tm*_slco2a1_* [°C] |
| --- | --- | --- |
| Experiment 1 | 77.83 | 80.44 |
| Experiment 2 | 77.63 | 80.17 |
| Experiment 3 | 77.59 | 80.25 |
| Experiment 4 | 77.61 | 80.29 |
| Mean | 77.67 | 80.29 |
| Standard deviation | ± 0.10 | ± 0.10 |

_Tm = melting temperature_

**Table S4:** Detection of melting temperatures of co-amplified fragments of nusG and slco2a1.

|  | Tm*_nusG_* [°C] | Tm*_slco2a1_* [°C] |
| --- | --- | --- |
| Experiment 1 | 76.61 | 80.02 |
| Experiment 2 | 76.69 | 80.24 |
| Experiment 3 | 76.69 | 80.24 |
| Mean | 76.66 | 80.17 |
| Standard deviation | ± 0.04 | ± 0.10 |

_Tm = melting temperature_

**Table S5:** Mean values of melting temperatures for slco2a1 and nusG.

|  | | Co-amplification | | Single amplification |
| --- | --- | --- | --- | --- |
|  |  | Tm*_slco2a1_* [°C] | Tm*_nusG_* _(samples)_ [°C] | Tm*_nusG_* _(PC)_ [°C] |
| Displayed data refers to values within each groupo | Group 1 (control) | 79.97 |  |  |
|  | Group 2 (UC) | 80.29 | 76.05 |  |
|  | Group 3 (LGD) | 80.59 | 76.68 |  |
|  | Group 4 (HGD) | 80.52 | 76.54 |  |
|  | Group 5 (CAC) | 80.39 | 76.09 |  |
|  | Group 6 (sCRC) | 80.29 | 76.09 |  |
| Mean (all groups) | | 80.34 | 76.29 |  |
| Standard deviation (all groups) | | ± 0.20 | ± 0.27 |  |
| Displayed data refers to mean values of all samples | Mean (all samples) | 80.32 | 76.21 | 77.71 |
|  | Standard deviation (all samples) | ± 0.33 | ± 0.53 | ± 0.17 |

_Tm = melting temperature; PC = positive control_
